# Functional 3D Human Primary Hepatocyte Spheroids Made by Co-Culturing Hepatocytes from Partial Hepatectomy Specimens and Human Adipose-Derived Stem Cells

**DOI:** 10.1371/journal.pone.0050723

**Published:** 2012-12-07

**Authors:** Da Yoon No, Seung-A Lee, Yoon Young Choi, DoYeun Park, Ju Yun Jang, Dong-Sik Kim, Sang-Hoon Lee

**Affiliations:** 1 Department of Biomedical Engineering, Korea University, Seoul, Republic of Korea; 2 Réal Aesthetic Plastic Surgery Clinic, Seoul, Republic of Korea; 3 Department of Surgery, Korea University, Seoul, Republic of Korea; University of Kansas Medical Center, United States of America

## Abstract

We have generated human hepatocyte spheroids with uniform size and shape by co-culturing 1∶1 mixtures of primary human hepatocytes (hHeps) from partial hepatectomy specimens and human adipose-derived stem cells (hADSCs) in concave microwells. The hADSCs in spheroids could compensate for the low viability and improve the functional maintenance of hHeps. Co-cultured spheroids aggregated and formed compact spheroidal shapes more rapidly, and with a significantly higher viability than mono-cultured spheroids. The liver-specific functions of co-cultured spheroids were greater, although they contained half the number of hepatocytes as mono-cultured spheroids. Albumin secretion by co-cultured spheroids was 10% higher on day 7, whereas urea secretion was similar, compared with mono-cultured spheroids. A quantitative cytochrome P450 assay showed that the enzymatic activity of co-cultured spheroids cultured for 9 days was 28% higher than that of mono-cultured spheroids. These effects may be due to the transdifferentiation potential and paracrine healing effects of hADSCs on hHeps. These co-cultured spheroids may be useful for creating artificial three-dimensional hepatic tissue constructs and for cell therapy with limited numbers of human hepatocytes.

## Introduction

The number of patients with chronic liver disease or cirrhosis is increasing continuously, accounting for 9.9 deaths per 100,000 population in the U.S. in 2009 [1]. Liver transplantation is the only long-term option for patients with end-stage liver disease, but is limited by the shortage of donors, its high cost, the high rate of rejection, and potential risks for living donors. Human liver-cell-based therapies, such as the bio-artificial liver and hepatocyte transplantation, may be an alternative to liver transplantation, decreasing the mortality rate of patients on the waiting list. Although several types of bio-artificial liver have been described, most are extracorporeal systems for short term use and their clinical success has been limited [2]. Hepatocyte-based therapies are less invasive than whole-organ or partial liver transplantation, with the possibility of repetition, and do not require chronic immunosuppression [3]. Hepatocyte transplantation, however, requires a continuous supply of human primary hepatocytes and their *in vitro* culture with functional maintenance for a period of time. To date, most primary human hepatocytes have been isolated from the livers of multi-organ donors (MODs) who were rejected from a transplantation program due to histological impairment or from liver specimens obtained during partial hepatectomy [4]. Although primary human hepatocytes from partial hepatectomy specimens are more reliable and useful sources, their viability is poor (40–80%), resulting in the more frequent use of hepatocytes from MODs [5], [6]. However, obtaining MODs are as cumbersome as entire transplantable livers. Therefore, increasing the number and viability of primary hepatocytes from partial hepatectomy specimens, and improving their functional maintenance, are of great importance for successful hepatocyte transplantation.

We describe here a novel method for using primary human cells from partial hepatectomy specimens to overcome these obstacles, including cell shortage and low viability. The key feature of this method is the generation of spheroids by co-culturing primary human hepatocytes (hHeps) and human adipose-derived stem cells (hADSCs) in concave microwell arrays. Culturing of hepatocytes as spheroids, which enhances the vitality and maintenance of hepatic performance, has been shown to improve their viability and function [7], [8]. Human adipose tissue has been shown to contain mesenchymal stem cell populations and hADSCs [9], [10]. The culture of hADSCs in media supplemented with growth factors and cytokines has been found to yield cells with hepatocyte-specific functions, including albumin production and urea synthesis [11], [12]. Based on these findings, we co-cultured both cells in 3D spheroid expecting a positively compensating effect. Such 3D co-culturing dramatically enhanced the viability of hHeps from partial hepatectomy specimens, spheroid formation with uniform shape and hepatic function, and enabled hADSCs to be transdifferentiated into hepatocyte-like cells without additional processes. These uniform-sized co-cultured spheroids may be useful sources for human liver transplantation and human hepatocyte cell therapy. Furthermore, they might be used to create 3D human liver models and to facilitate studies of human liver physiology and pathophysiology, as well as contributing to the recovery of failed human liver function and the development of bioartificial liver systems, overcoming cell shortage problems.

## Materials and Methods

### Isolation and culture of primary hHeps and hADSCs

Human hepatocytes were isolated under sterile conditions from the healthy resection margins of liver samples obtained during partial hepatectomy, using a two-step collagenase perfusion technique [13]. Briefly, liver tissue was perfused at 37°C with ethylene glycol-bis(β-aminoethyl ether)-N,N-tetraacetic acid (EGTA), followed by 0.1% collagenase at a flow speed that depended on the size of the liver specimen. The digested liver tissue was placed in cold Leffert's buffer, the liver capsules were cut, and cells were dissociated. Hepatocytes were mobilized by gentle shaking, and the suspension was filtered through a nylon mesh of pore size 70 µm. The cell suspension was centrifuged 3 times at 50×g for 5 minutes each at 4°C to separate the hepatocytes from the nonparenchymal cells. Hepatocyte viability and concentration were determined using the Trypan blue exclusion technique; the average viability of isolated hepatocytes was 62% (Table 1). Primary human hepatocytes were cultured in high-glucose Dulbecco's Modified Eagle's Medium (DMEM) supplemented with 20 mM HEPES (Sigma Aldrich), 25 mM NaHCO_3_, 30 mg/L-proline, 10% fetal bovine serum (FBS), 25 U/ml penicillin, 25 µg/ml streptomycin, 10 µg/ml gentamicin, 10 ng/ml epidermal growth factor (EGF), 50 ng/ml insulin, 10^−4^ M dexamethasone, 10 mM nicotinamide, and 100 mM L-ascorbic acid. hADSCs were isolated from leftover human subcutaneous adipose tissue of patients undergoing plastic surgery or liposuction, as described [14]. Isolated hADSCs were cultured and expanded at 37°C in low-glucose DMEM containing 10% FBS, 50 U/ml penicillin and 50 µg/ml streptomycin, in an atmosphere containing 5% CO_2_. All procedures adhered to the guidelines of the IRB of Korea University. Written informed consent was obtained from all patients, and the protocol of this study was approved by the hospital ethics committee.

**Table 1:** Isolation of primary human hepatocytes from partial hepatectomy specimens.

Specimen	Weight (g)	Cell Yield (×10^6^)	Viability (%)
A	27.2	7.0	67
B	32	25	60
C	26.4	10	75
D	30	14	46
E	18.4	15	47
F	19.97	2.7	52
G	19.07	30	70
H	18.29	54	56
I	28.9	45	64
J	13.4	6.2	69
K	19.8	23	52
L	27	3.0	50
M	18.27	9.8	81
N	12.9	25	76
O	11.48	15	60
P	26.38	34	56
Q	18.46	7.9	73

### Formation of mono- and co-cultured spheroids

**Figure 1:**
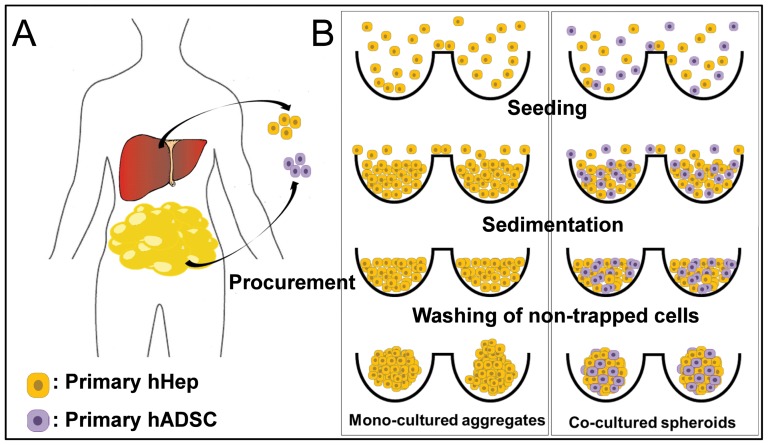
Schematics procedures of mono- and co- cultured spheroid formation. hHeps were isolated from the healthy resection margins of liver samples obtained during partial hepatectomy, and hADSCs were isolated from leftover human subcutaneous adipose tissue of patients undergoing plastic surgery or liposuction. Cells were seeded onto concave microwells and cultured for a few days to form cell aggregates and spheroids.

Spheroids were generated using poly(dimethylsiloxane) (PDMS)-based concave micromolds developed using thin PDMS membranes [15]–[17]. Concave micromolds of 500 µm diameter were fabricated using soft lithography techniques. The concave microwells were coated with 3% (w/v) bovine serum albumin (BSA) overnight to prevent cell attachment. Human hepatocytes were seeded onto these wells and cultured for a few days to form mono-cultured spheroids. To form co-cultured spheroids, the wells were seeded with a 1∶1 mixture of human hepatocytes and hADSCs and the cells were cultured for a few days (Fig. 1). Cell aggregation and spheroid formation were observed daily under a microscope.

### Scanning electron microscopy (SEM)

Spheroids formed in concave microwells were fixed with 2.5% glutaraldehyde in deionized water for 1 h and gently washed 3–5 times with deionized water. For secondary fixation, spheroids were immersed in 1% osmium tetroxide in deionized water for 1–2 h. The fixed spheroids were subsequently dehydrated with a graded ethanol series (25%, 50%, 75%, 95%, and 100%), immersed in tert-butyl alcohol three times for 30 min each at room temperature and frozen at −70°C. The spheroids were freeze-dried until the tert-butyl alcohol had evaporated, mounted onto a specimen stub with graphite paste, coated with palladium alloy, and observed under a scanning electron microscope (JEOL Ltd, Tokyo, Japan).

### Transmission electron microscopy (TEM)

Spheroids formed from hHeps and hADSCs in concave PDMS microwells for 7 days were fixed in a solution containing glutaraldehyde. The samples were sliced and observed under a transmission electron microscope (JEOL, Tokyo, Japan) operated at 80 kV.

### Immunofluorescence staining

Cells were fixed with 4% paraformaldehyde (PFA) for 30 min at 4°C and incubated in 0.1% Triton X-100 in phosphate-buffered saline (PBS) for 20 min at room temperature. After rinsing with 0.1% BSA in PBS, the cells were incubated with Block Ace (Dainippon Pharma, Tokyo, Japan) at 4°C for 30 min and subsequently incubated overnight at 4°C with rabbit antibodies to serum albumin (Santa Cruz Biotechnology Inc, USA) and cytochrome P450 reductase (Abcam, UK). The cells were rinsed with 0.1% BSA in PBS and incubated at 4°C for 90 min with Alexa Fluor 488-conjugated or Alexa Fluor 594-conjugated anti-rabbit IgG (Invitrogen) secondary antibodies, as appropriate. The cells were then incubated with DAPI (40,6-diamidino-2-phenylindole) for 5 min at room temperature, and confocal microscopic images were obtained (Olympus, Japan).

### Cell viability

Cell viability was assessed by incubating spheroids with 50 mM Calcein-AM and 25 mg/ml ethidium homodimer-1 (EthD-1; Molecular Probes, USA) in culture medium for 40 min at 37°C, followed by imaging under a light microscope.

### Functional assessment

Albumin and urea secretion were analyzed by measuring the concentrations of albumin and urea in medium conditioned by cultured spheroids. Spheroids were cultured in concave PDMS microwells for 1, 3, 5 and 7 days; at each time point, 300 µl of medium was removed and replaced with 300 µl of fresh medium. Cytochrome P450 3A4 (CYP3A4) enzymatic activity was determined using the luminescent P450-Glo CYP3A4 Cell-based Assay (Luciferin-PFBE) (Promega), as described by the manufacturer. Briefly, mono- or co-cultured spheroids were incubated with luminogenic substrate (Luciferin-PFBE) in hepatocyte culture medium for 3–4 h at 37°C. The medium was subsequently transferred to a 96-well opaque white plate, and an equal volume of Luciferin Detection Reagent was added to initiate a luminescent reaction. The plate was incubated at room temperature for 20 min, and luminescence was read using a Multimode Plate Reader (PerkinElmer).

### Gene analysis

Co-cultured spheroids in concave microwells were gently retrieved after 3 and 7 days. Total RNA was isolated from these cell preparations, as well as from freshly isolated hepatocytes, using TRIzol reagent (Invitrogen, CA), chloroform extraction and precipitation with isopropyl alcohol. cDNA was synthesized from the purified RNA using reverse transcriptase (TAKARA, Japan) as per the manufacturer's instructions.

## Results

**Figure 2:**
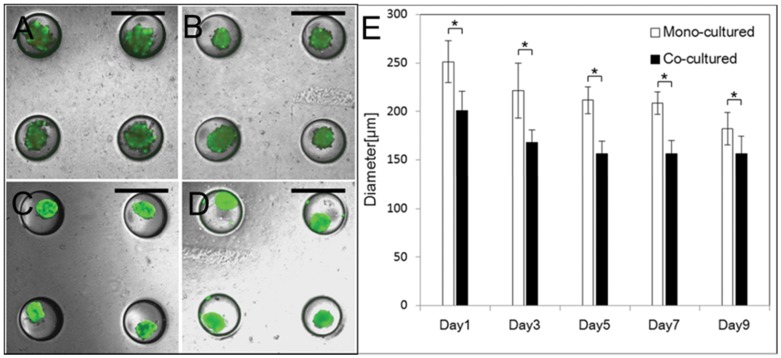
Co-cultured spheroids had higher viability and aggregated more rapidly than mono-cultured spheroids. Spheroidal formation and viability of cells in mono-cultured spheroids cultured for (A) 3 days, (B) 7 days and in co-cultured spheroids cultured for (C) 3 days and (D) 7 days in concave microwells. Scale bars, 500 µm, (E) Diameter analysis of mono-cultured spheroids and co-cultured spheroids in 500 µm concave microwells. Data represent the means ± standard deviations of 10 independent experiments (*p<0.0001, two-tailed test).

As shown in Fig. 1A, hHeps were isolated from human liver specimens after partial hepatectomy, and details for specimen are summarized in Table 1. hADSCs were from leftover human subcutaneous adipose tissue of patients undergoing plastic surgery or liposuction. We seeded prepared cells in concave microarray wells, which has been an excellent cell spheroid production platform for rodent hepatocytes, β-cells and stem cells [15]–[17], to generate uniformly-sized and shaped spheroids (mono-culture of hHeps and 1∶1 co-culture of hHeps and hADSCs) (Fig. 1B). The characteristics of mono-cultured and co-cultured spheroids, including their size, shape, and viability, were assessed quantitatively over time. Contrary to rodent hepatocyte cells, [17] the mono-culture of hHeps did not generate well-organized ultra-structures. Although sufficient hHeps were seeded on concave microwells, they were hardly aggregated and did not form uniform-shaped spheroids until they were cultured for 7 days because of the poor condition of hHeps from partial hepatectomy specimens. In contrast, the 1∶1 co-culture of hHeps and hADSCs created stable and uniformly-sized spheroids one day after the seeding (Fig. 2). Cell viability in mono-cultured and co-cultured spheroids after 3 and 7 days was 47% vs 99% and 64% vs 99%, respectively (Fig. 2A–D). Magnified fluorescent images of single mono-cultured and co-cultured spheroids on day 9 show that long-term culture and the retrieval process did not affect cell viability (Fig. S1).

To compare the size of mono-cultured and co-cultured spheroids quantitatively, we measured their diameters over time (Fig. 2E). Following culture for 1 day in 500 µm microwells, their average sizes were reduced to 250 µm and 200 µm, respectively, and decreased to 225 µm and 170 µm, respectively, on day 3. The size of co-cultured spheroids remained almost constant through days 5–9, in contrast to the size of mono-cultured spheroids, which decreased. These results show that co-cultured spheroids aggregated more rapidly and formed a tighter spheroidal shape than mono-cultured spheroids.

**Figure 3:**
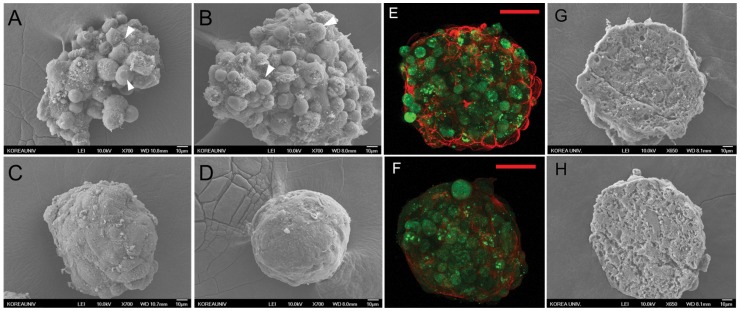
Only co-cultured model can form compact spheroidal shape. SEM images of mono-cultured spheroids cultured for (A) 3 days, (B) 9days and co-cultured spheroids cultured for (C) 3 days and (D) 9 days. Scale bars, 10 µm. Immunostaining for AE-2 (green) and actin (red) in (E) mono-cultured spheroids and (F) co-cultured spheroids cultured for 9 days. Scale bars, 50 µm. Scanning electron microscopy images of sectioned co-cultured spheroids cultured for (G) 3 days and (H) 9days. Inner structure of co-cultured spheroids is porous which involves good diffusion of nutrient and oxygen into the spheroid. Scale bars, 10 µm.

SEM images clearly showed that co-cultured spheroids aggregated more tightly and rapidly than mono-cultured spheroids and had smoother surfaces (Fig. 3A–D). Mono-cultured spheroids on day 3 lost their original shape during SEM preparation due to loose cell-cell interaction. In contrast, the aggregates of co-cultured spheroids became denser and had smoother surfaces over time. Immunostained images of the actin in spheroids clearly demonstrate the tight cell-cell interaction on the surface (Fig. 3E and F). Mono-cultured spheroids had cell-like round surfaces, whereas co-cultured spheroids had smooth surfaces demonstrating tight cell-cell interaction. SEM images of cryosectioned spheroids co-cultured for 3 and 9 days showed tight porous structures and no necrosis was observed (Fig. 3G and H).

**Figure 4:**
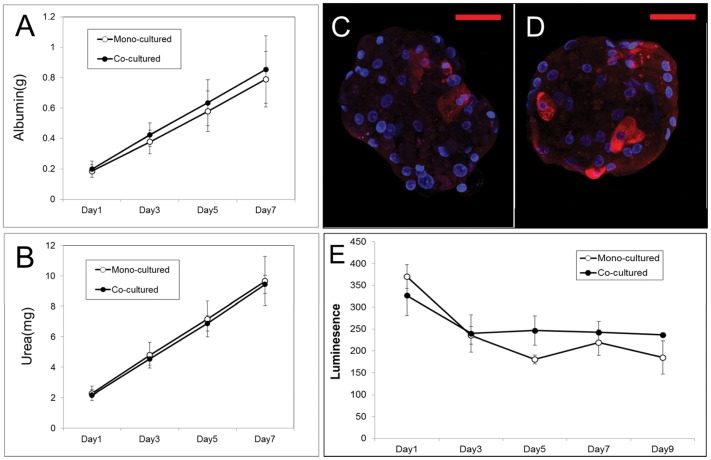
Co-cultured spheroids showed higher liver specific function and more stable metabolic function than mono-cultured spheroids. Analysis of metabolic function of mono-cultured spheroids and co-cultured spheroids, measured as secretion of (A) albumin and (B) urea. Data represent means ± standard deviations of eight independent experiments. Immunostaining for cytochrome P450 reductase (red) in (C) mono-cultured spheroids and (D) co-cultured spheroids cultured for 9 days. Nuclei were stained with DAPI (blue). Scale bars, 50 µm. (E) Luminescence assay of cytochrome P450 activity in mono-cultured and co-cultured spheroids. Cytochrome P450 3A enzyme activity were measured based on the degree of luminescence emitted by adding detection reagent and measured with a luminometer. Data represent means ± standard deviations of 3 independent experiments.

We analyzed the liver specific functions of these spheroids by measuring albumin and urea secretion. Quantitative analysis showed that the co-cultured spheroids secreted about 10% more albumin than mono-cultured spheroids, although the former contained only about half the number of hHeps (Fig. 4A); urea secretion by co-cultured and mono-cultured spheroids was similar (Fig. 4B). The immunostained image also showed that the human albumin was expressed strongly on co-cultured spheroids, despite half the cells being hADSCs (Fig. S2).

We analyzed the metabolic activity of hepatocytes, by staining with cytochrome P450 reductase and by monitoring CYP3A4 activity over 9 days. The results showed that the expression of cytochrome P450 reductase (red) was greater in the exterior cells of co-cultured than that of mono-cultured spheroids (Fig. 4C and D). In addition, a luminescence assay showed higher and more stable CYP3A4 enzymatic activity in co-cultured than in mono-cultured spheroids (Fig. 4E).

**Figure 5:**
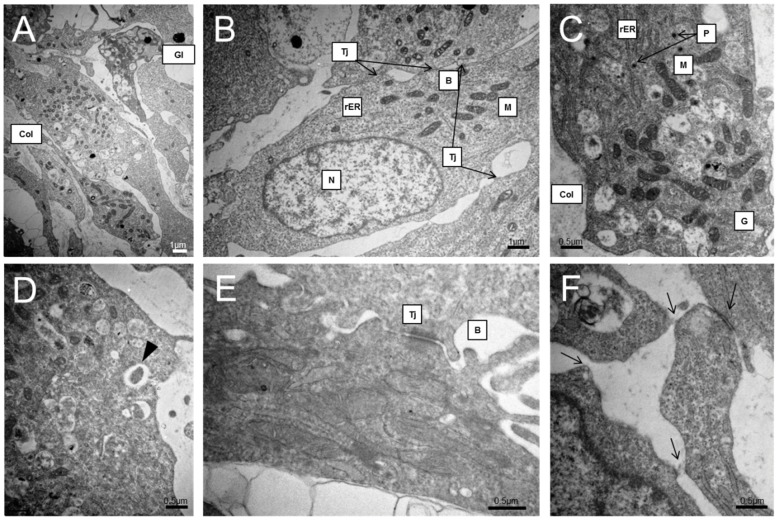
Co-cultured spheroids showed healthy hepatocytic ultrastructure. Ultrastructural feature of the co-cultured spheroids by transmitting electron microscopy (TEM) on day 7 of culture. The spheroids are characterized by tight junctions (Tj) between adjacent cells, distinct nuclei (N), abundant mitochondria (M), peroxisomes (P), rough endoplasmic reticulum (rER), collagen accumulation (Col), glycogen vesicles (Gl), and bile canaliculi (B).

Seven days after seeding, we examined the ultrastructure of co-cultured spheroids formed within concave microwells by TEM (Fig. 5). These spheroids displayed multi-cell layer morphology with elongated cell features and tight junctions (Tj) between adjacent cells, emphasizing close cell-cell interactions. The cells in spheroids exhibited distinct nuclei (N), abundant mitochondria (M), rough endoplasmic reticulum (rER), and glycogens (Gl) (Fig. 5A–C). Furthermore, these cells displayed polarity, as shown by the presence of bile canaliculi (B) possessing microvilli, bordered by tight junctions (Fig. 5E) [18]. Similar features have been observed in spheroids formed by primary rat hepatocytes [19] and human hepatocyte cell lines [20]. Abundant peroxisomal (P) vesicles are often present, as is the accumulation of extracellular matrix, such as collagen, over a broad area (Fig. 5C). Compared with TEM images of hADSCs cultured for 7 days alone (Fig. S3), hADSCs in co-cultured spheroids exhibited different features and looked more similar to hHeps than hADSCs. These results suggest that hADSCs might be transdifferentiated to hepatocyte-like cells.

**Figure 6:**
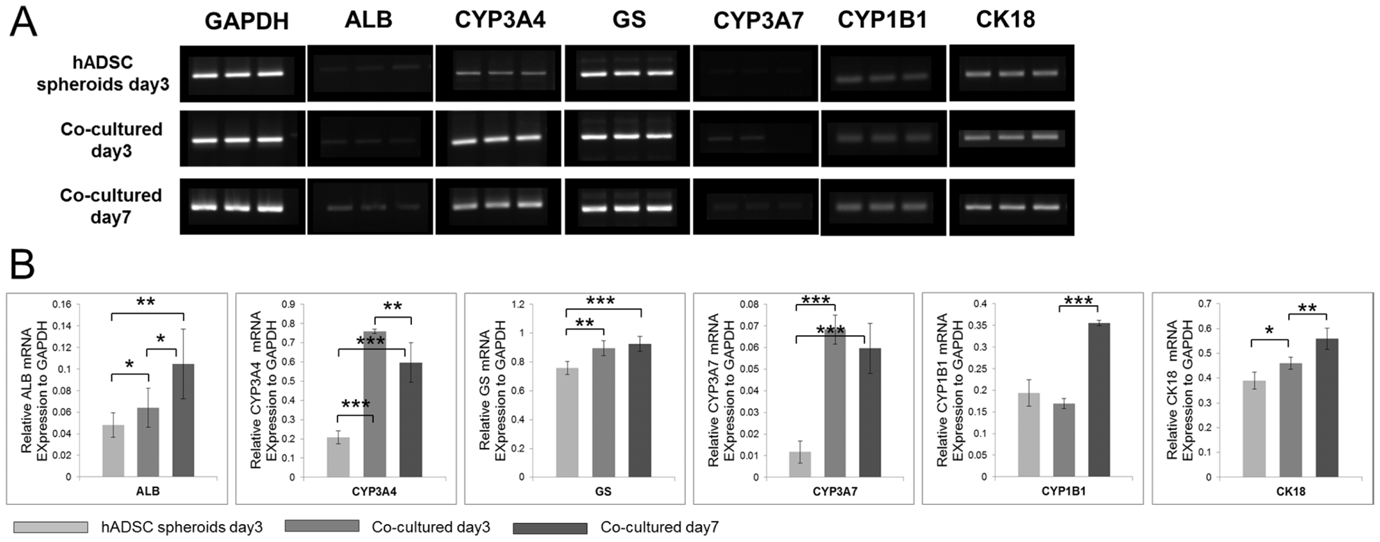
hADSCs in co-cultured spheroids exhibited hepatocyte-like features. (A) The expression of mRNA for the co-cultured spheroids on day 3, day 7, and hADSC mono-cultured spheroids on day3. (B) Quantification of the relative gene expressions to GAPDH. Data represent the means ± standard deviations of 3 independent experiments. (*p<0.1, **p<0.05, ***p<0.001, two-tailed test).

To determine whether hADSCs within the co-cultured spheroids express hepatocyte-like features, we assayed the mRNA expression levels of CYP3A7, a marker of immature hepatocytes, and ALB, CYP3A4, glutamine synthetase, CYP1B1 and CK18, which are markers of mature hepatocytes, in co-cultured spheroids on days 3 and 7, and in hADSC spheroids on day 3 (Fig. 6). The mRNA expression levels of all markers became higher in co-cultured spheroids than in hADSC spheroids as time passed, which indicates that hADSCs in co-cultured spheroids exhibited hepatocyte-like features.

## Discussion

We have shown here that uniformly-sized and shaped hHeps and hHeps-hADSCs spheroids could be successfully generated in concave microwell arrays. Because producing uniform 3D hepatocyte spheroids are very important, several approaches have been previously reported to date, including non-adherent surface, matrix array, and rocked culture. However, the development of well-controlled 3D culture systems that can produce large amounts of size-controlled uniform spheroids has remained a challenge. In the concave microarray wells that were used in this paper, we could overcome all these obstacles and achieved the production of large amounts of size-controllable uniform spheroids very easily and rapidly. Moreover, these spheroids were detached easily from the wells, without the need for treatment with trypsin or collagenase due to the concave-morphology effect [17], and this retrieval process did not affect cell viability at all. This kind of polymer based microwells also could be applied as useful tools in embryoid body (EB) formation instead of conventional methods like hanging-drop and suspension culture [16], [21]. The 3D co-culture of hHeps and hADSCs improves the spheroid formation and functional maintenance of hHeps so it can compensate for the lack of hHeps. To date, there have been a few reports describing the use of hADSCs in a 2D culture environment for the functional improvement of human hepatocytes [22], however, a 3D co-culture model is very rare. Moreover, the quantitative evaluation of 3D co-culture effects between hHeps and hADSCs may be the first to our knowledge. Our 3D co-culture model showed the positive effects of hADSCs on hepatocyte function and spheroid formation, which might be the first of its kind. We found that the mono-cultured hHeps spheroids did not show cellular aggregation until day 9, demonstrated by individual cells at the spheroid surface (Fig. 3A and B white arrows). In contrast, the mixture of hHeps and hADSCs was aggregated rapidly, forming compact spheroids in concave microwells by day 3, which indicates that hADSCs could play a key role in spheroid formation of hHeps similar to hepatic-stellate cells [17]. SEM images of mono- and co-cultured spheroids clearly demonstrated the effects of hADSCs on compact spheroid formation. The size of co-cultured spheroids decreased drastically after cell seeding, which might be due to tight cell-cell interactions. Though initial viability of hHeps was diverse between liver specimen samples, the viability of co-cultured spheroids was much higher than that of mono-cultured spheroids regardless of sample diversity. Gimble *et al.* reported that hADSCs transferred into a site of tissue injury or disease may secrete cytokines and growth factors that stimulate recovery in a paracrine manner by stimulating the activity of antioxidant chemicals, free radical scavengers, and chaperone/heat shock proteins [10]. Such wound healing effects of hADSCs may be related to the improvements in poor viability and spheroid formation of human hepatocytes. The interior structures of co-cultured spheroids were imaged by the cryosectioned spheroids cultured for 3 and 9 days, revealing that the interiors of both spheroids were porous. Nutrients and gases could easily diffuse through these pores, potentially accounting for the absence of central necrosis, even in spheroids over 170 µm in diameter.

The hepatic functions of spheroids were evaluated by assaying their secretion of albumin and urea over 7 days. Both types of spheroids secreted albumin and urea continuously, yet, co-cultured spheroids secreted a little greater quantity of albumin, despite having only half the number of hHeps as the mono-cultured system. Immunodetection of cytochrome P450 reductase and analyses of the enzymatic activity of CYP3A4 showed that the levels and activity of this enzyme, one of the most important for the metabolism of xenobiotics, were comparatively higher in co-cultured than in mono-cultured spheroids. Although the absolute functional activities of human hepatocytes vary among liver samples, we found that co-cultured spheroids generally functioned better than mono-cultured spheroids. These results indicate that hepatic function and phenotype were better maintained in our 3D model.

TEM images showed well-developed hepatic ultrastructural characteristics. The spheroids maintained their viability and function for 1 week in culture, and demonstrated the extensive appearance of metabolism-related organelles, including mitochondria, rER, peroxisomes, and endocytosis or exocytosis (Fig. 5D black arrow), which indicates that metabolic functions were well preserved in 3D spheroidal culture. They were also making a number of new tight junctions (Fig. 5E skeletal arrows), indicating that hepatocytic interaction was conducted continuously. Although the SEM image of the co-cultured spheroid showed tight junctions, the cell membranes were not closely attached to each other, with many non-cellular spaces occupied by ECM-like materials (Fig. 5A and C, Col). These non-cellular spaces may play a role in exchanging nutrients and gases. Although these spheroids were co-cultured with hADSCs, they exhibited hepatocytic ultrastructural features, rather than hADSCs'. From these experimental results, we expected that the reason for the high performance of co-cultured spheroids, despite having half the number of hepatocytes, may be from the differentiation of hADSCs into mostly hepatocyte-like cells in co-cultured spheroids, which can improve hepatocytic functions and features. To date, several researchers have reported that hepatocyte-like cells can be generated from hADSCs by the sequential addition of growth factors *in vitro* for the transdifferentiation of hADSCs into hepatocyte-like cells [11], [23], [24]. Only at high density could fetal hepatic cells and MSCs be induced to form cells with a hepatocyte phenotype [25]. Furthermore, adult hepatocyte progenitor cells were found to express high levels of mature hepatocyte markers when cultured in spheroids [22], [26]. Based on these studies, we can conclude that our spheroidal model could provide the adequate environment for hADSCs to transdiffrentiate into hepatocyte-like cells via high cell density and the tight cell-cell interaction feature of spheroids. For the proof of this hypothesis, gene-based studies were performed. The co-cultured spheroids expressed high levels of immature and mature hepatocyte markers, including CYP3A7, ALB, CYP3A4, glutamine synthetase, CYP1B1 and CK18 though they consisted of 50% hADSCs. Human MSCs in cell pellets have been shown to differentiate into hepatocyte-like cells that express a subset of hepatic genes and in which CYP3A4 mRNA is inducible. However, mono-cultured hADSCs spheroids exhibited lower levels of hepatocytic gene expression compared to the co-cultured model, indicating that hepatocytic gene expression of hADSCs may be enhanced by not only a spheroidal shape, but also co-culture with hHeps and direct cell-cell interaction.

In summary, we discovered that hADSCs in co-cultured 3D spheroids assist in the formation of compact hepatic spheroids in concave microwell arrays, enhance the viability of hepatocytes even from partial hepatectomy, and behave like hepatocytes. The shortage of donor hHeps is a serious obstacle for successful cell-based liver regeneration, whereas hADSCs, which can be repeatedly obtained in large quantities under local anesthesia, may be a promising cell source for clinical use [27]. Co-cultured spheroids may provide a new therapeutic modality as a cell transplantation paradigm for end-stage or acute liver failure patients. In addition, these co-cultured spheroids could be used in extracorporeal bioartificial livers. Furthermore, a 3D co-cultured spheroid model might be used for the *in vitro* liver model for the study of interaction between hepatocyte and non-parenchymal cells. As hADSCs can be differentiated toward osteogenic, adipogenic, neurogenic, myogenic and chondrogenic lineages, co-culture with these cells may be a useful model in characterizing their differentiation property into cells of a specific lineage. Furthermore, this 3D spheroid model, combined with microfluidic technology, may be used for drug-screening or high throughput hepatotoxicity tests.

## Conclusions

In this paper, we proposed that the hADSCs in 3D spheroid co-cultured with hHeps assist the compact formation of hepatic spheroids in concave microwell arrays and behave like hepatocytes. The co-cultured spheroids might facillitate a more efficient, easier, and safer method than whole organ transplantation to cure patients suffering from end-stage liver disease, overcoming the shortage of donor hHeps. In addition, they could be used in the extracorporeal bioartificial liver maintaining stable cell viability and functions. However, further *in vitro* and *in vivo* studies are needed to achieve a better understanding of the potential benefits and risks of cell-based therapeutic usage in clinical settings.

## Supporting Information

Figure S1 **Confocal microscopic images of retrieved cell viability in (A) mono-cultured spheroids and (B) co-cultured spheroids on day 9.** Dead cells were stained red. Scale bars, 50 µm.
(TIF)

Figure S2 **Immunostaining for serum albumin (green) in (A) mono-cultured spheroids and (B) co-cultured spheroids cultured for 9 days.** Nuclei were stained with DAPI (blue). Scare bars, 50 µm.
(TIF)

Figure S3 **Ultrastructural feature of the hADSCs by transmitting electron microscopy (TEM) on day 7 of culture.** hADSCs are characterized by a large round cell body with numerous pseudopodia that stretched from cell surface. Cell nuclei were also relatively large and exhibited lobular or polygonal shapes. hADSCs cytoplasm contained a high quantity of mitochondria and ribosomes.
(TIF)

Table S1 Nucleotide sequences of gene-specific primers used for reverse transcription-PCR.
(DOCX)
