# Supplementary figures and images for: Functional 3D Human Primary Hepatocyte Spheroids Made by Co-Culturing Hepatocytes from Partial Hepatectomy Specimens and Human Adipose-Derived Stem Cells
Source: PLoS One. 2012 Dec 7;7(12):e50723. doi: 10.1371/journal.pone.0050723 (PMC3517565; doi:10.1371/journal.pone.0050723)

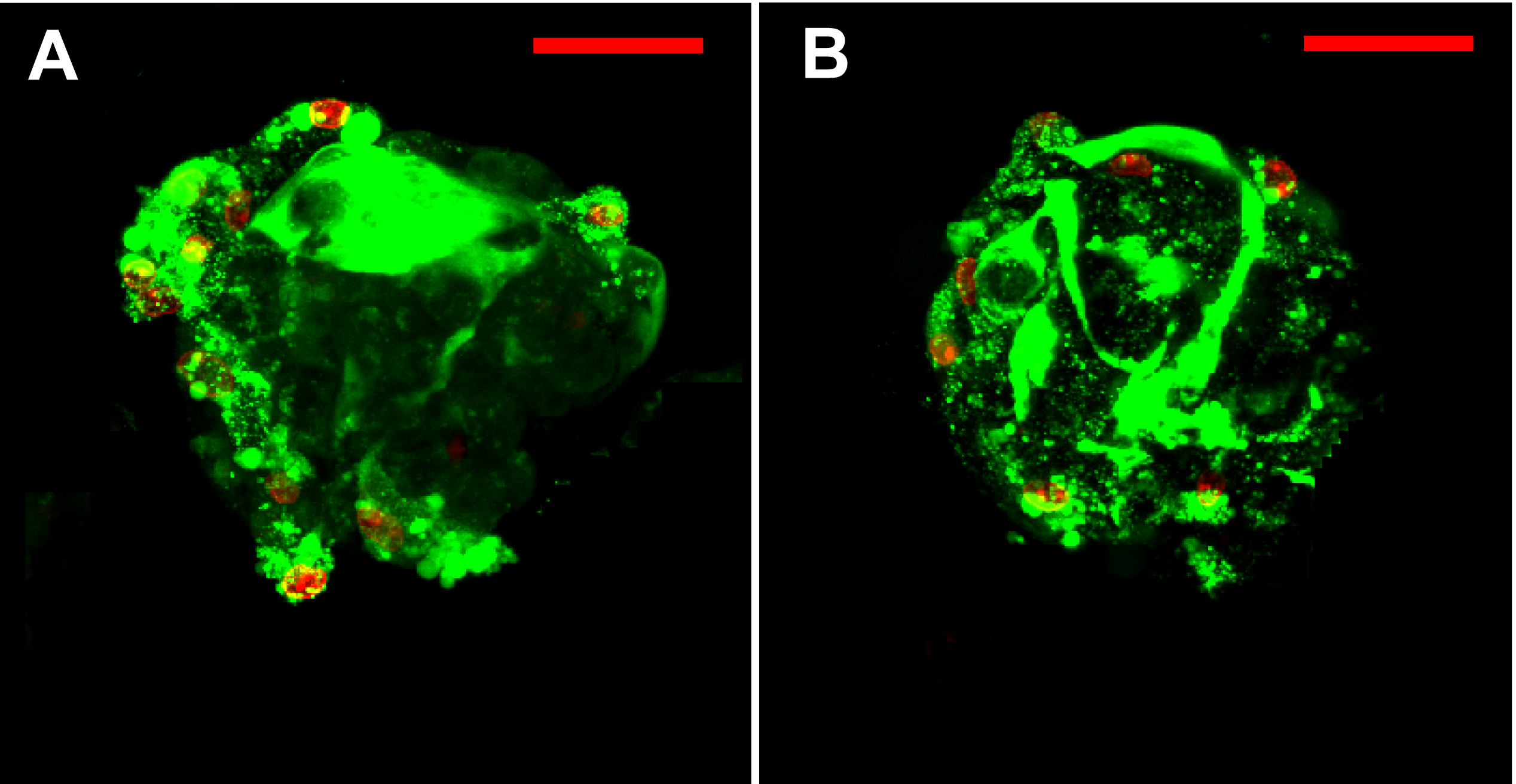

Supplement: Figure S1 — Confocal microscopic images of retrieved cell viability in (A) mono-cultured spheroids and (B) co-cultured spheroids on day 9. Dead cells were stained red. Scale bars, 50 µm. (TIF) [file pone.0050723.s001.tif]

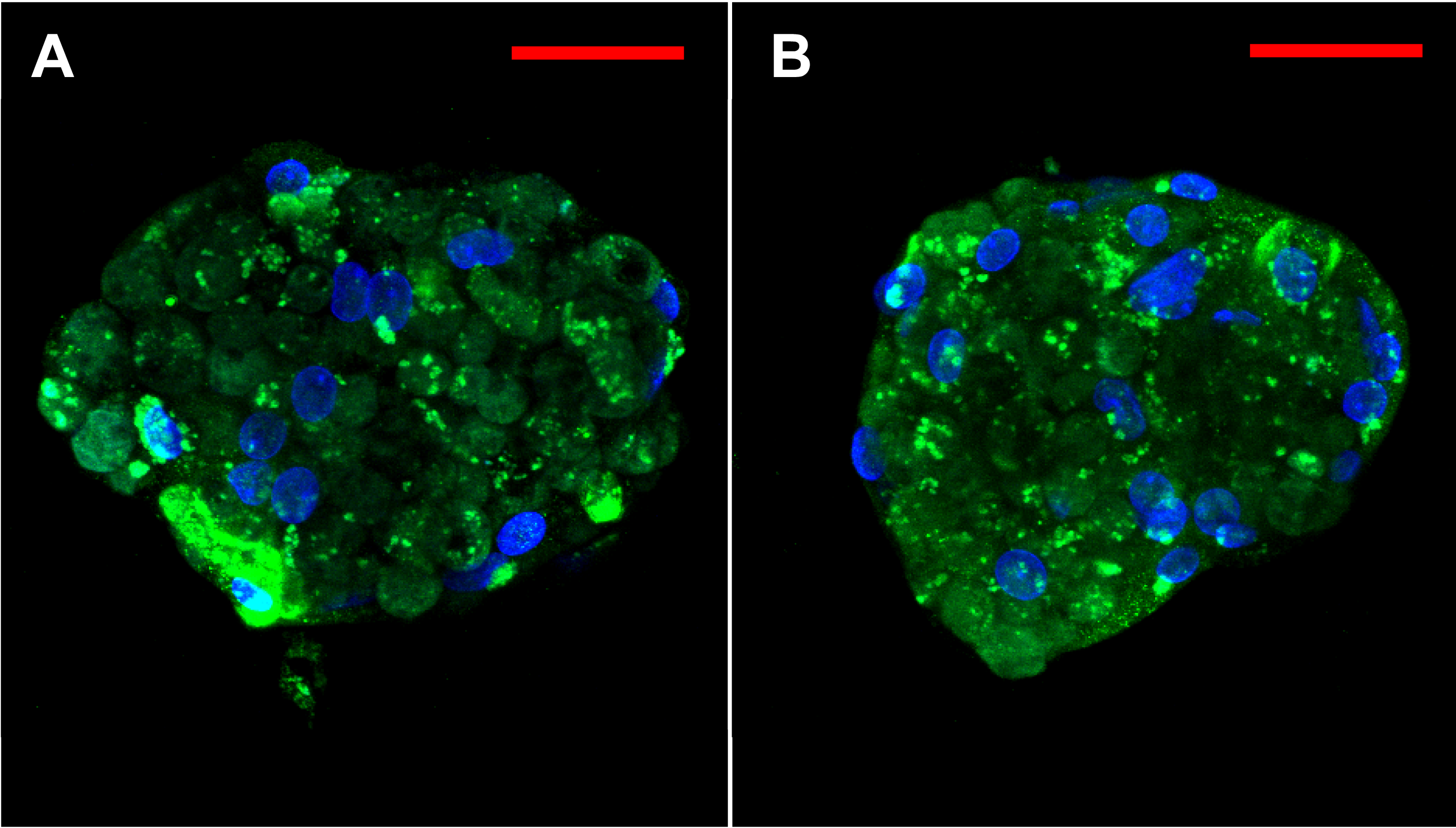

Supplement: Figure S2 — Immunostaining for serum albumin (green) in (A) mono-cultured spheroids and (B) co-cultured spheroids cultured for 9 days. Nuclei were stained with DAPI (blue). Scare bars, 50 µm. (TIF) [file pone.0050723.s002.tif]

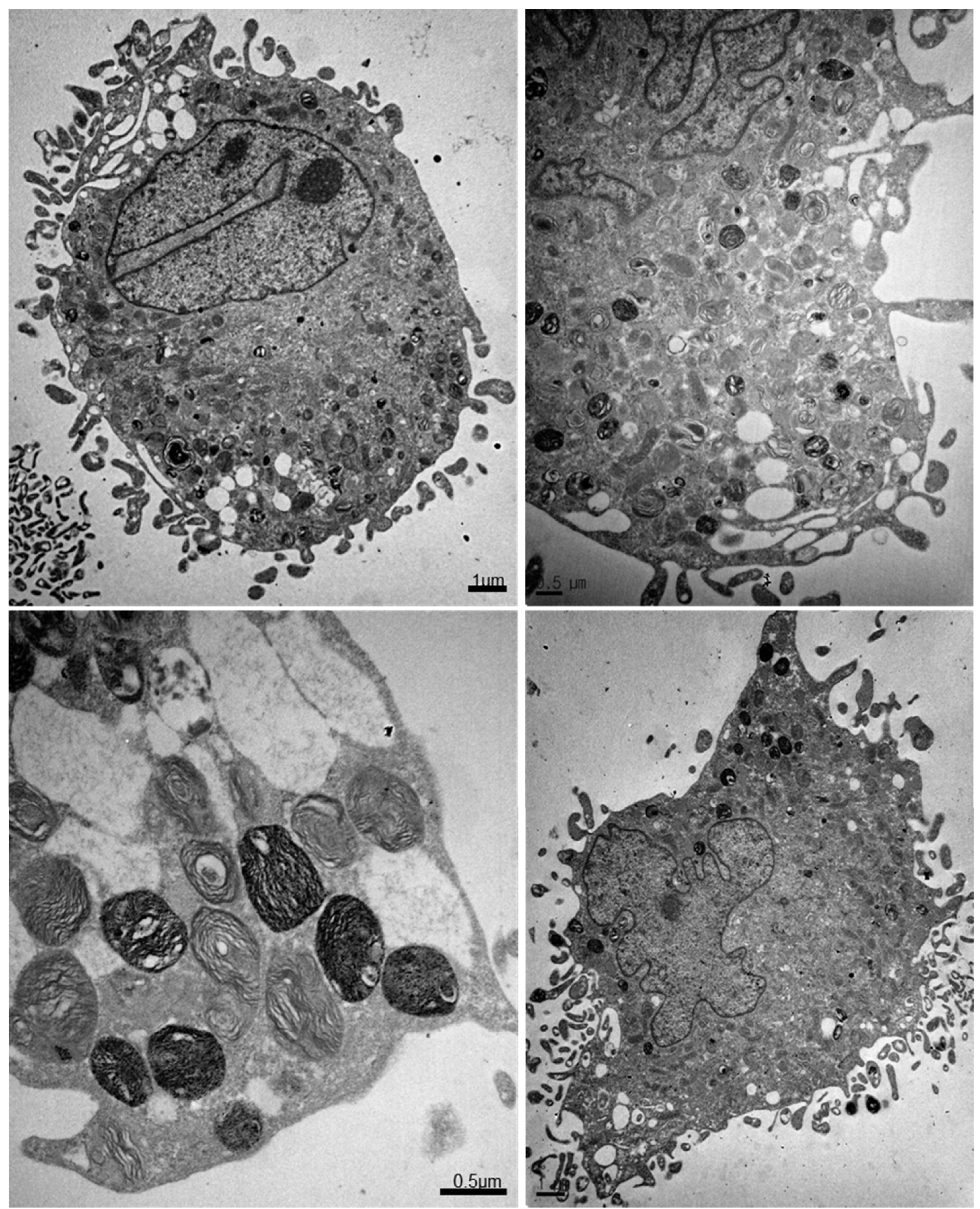

Supplement: Figure S3 — Ultrastructural feature of the hADSCs by transmitting electron microscopy (TEM) on day 7 of culture. hADSCs are characterized by a large round cell body with numerous pseudopodia that stretched from cell surface. Cell nuclei were also relatively large and exhibited lobular or polygonal shapes. hADSCs cytoplasm contained a high quantity of mitochondria and ribosomes. (TIF) [file pone.0050723.s003.tif]
