# Supplementary material for: Functional 3D Human Primary Hepatocyte Spheroids Made by Co-Culturing Hepatocytes from Partial Hepatectomy Specimens and Human Adipose-Derived Stem Cells
Source: PLoS One. 2012 Dec 7;7(12):e50723. doi: 10.1371/journal.pone.0050723 (PMC3517565; doi:10.1371/journal.pone.0050723)
Supplement: Table S1 — Nucleotide sequences of gene-specific primers used for reverse transcription-PCR. (DOCX) [file pone.0050723.s004.docx]

**Table S1.**

Nucleotide sequences of gene-specific primers used for reverse transcription-PCR

| **Gene** | **Sequences of forward and reverse primers (5’ to 3’)** |
| --- | --- |
| CYP1B1 | GCT GCA GTG GCT GCT CCT CCC ACG ACC TGA TCC AAT TCT |
| GS | GTC AAG ATT GCG GGG ACT AA TAC GAT TGG CTA CAC CAC CA |
| CK18 | GAG ATC GAG GCT CTC AAG GA CAA GCT GGC CTT CAG ATT TC |
| CYP3A4 | CAC AAA CCG GAG GCC TTT TG ATC CAT GCT GTA GGC CCC AA |
| CYP3A7 | AAG GGC TAT TGG ACG TTT GAC ATC CCA CTG GCC CGA AAG |
| GAPDH | AGC CAC ATC GCT CAG ACA CC GTA CTC AGC GGC CAG CAT CG |
